# Evaluating shear wave elastography as a predictor of extracorporeal shock wave lithotripsy outcomes in children

**DOI:** 10.1007/s00240-025-01796-z

**Published:** 2025-06-25

**Authors:** Mehmet Demir, İsmail Yağmur, Osman Dere, İbrahim Halil Albayrak, Abdulhakim Şengel

**Affiliations:** 1 Department of Urology, Harran University Faculty of Medicine, Sanliurfa, Turkey; 2 Department of Pediatric Urology, Harran University Faculty of Medicine, Sanliurfa, Turkey; 3 Department of Radiology, Harran University Faculty of Medicine, Sanliurfa, Turkey; 4 Department of Urology, Şanliurfa Training and Research Hospital, Sanliurfa, Turkey; 5 Department of Anhestesiology and Reanimation, Harran University Faculty of Medicine, Sanliurfa, Turkey

**Keywords:** Kidney stone, Extracorporeal shock-wave lithotripsy, Elastography, Hounsfield unite, Pediatric urolithiasis

## Abstract

The aim of this study was to demonstrate the utility of Shear Wave Elastography (SWE) in predicting the success of extracorporeal shock wave lithotripsy (ESWL) in pediatric patients. A total of 102 patients < 18 years of age with a diagnosis of kidney stones underwent ESWL between May 2021 and December 2023. SWE measurements of the stones were performed in all patients prior to ESWL. The subjects were divided into two groups: those who responded to ESWL and those who did not. Age, gender, stone location, stone size, body mass index (BMI), Hounsfield Unit (HU), and stone SWE values were compared between the groups. Among the 102 patients included in the study, 78 exhibited a positive response to ESWL. In the responder group, the SWE, HU, and stone size values were significantly lower than the non-responder group (*p* < 0.05). SWE exhibited significant efficacy in discriminating between responders and non-responders [area under the curve (AUC): 0.979 & *p* = 0.000]. A SWE cutoff value of 13.70 kPa was identified for patient differentiation [AUC:0.929 & *p* = 0.000]. SWE appears to be an effective method for predicting the success of ESWL in pediatric patients and may serve as an alternative parameter to HU for pre-treatment evaluation.

## Introduction

The incidence of pediatric urolithiasis is rapidly increasing worldwide, driven by changes in diet and lifestyle. This increasing trend poses significant clinical challenges and escalates healthcare costs [1]. Recent technological advances have shifted treatment modalities from open surgeries to minimally invasive procedures [2]. Among these, extracorporeal shock wave lithotripsy (ESWL) has emerged as the first-line treatment for kidney stones in children due to its low complication rates and high efficacy. Its advantages include outpatient applicability, effective sedation, repeatability, and a less invasive profile compared with other treatments [3].

Factors influencing the outcome of ESWL include the child’s age, stone size and location, stone hardness, body mass index (BMI), operator experience, and renal anatomy [4]. Of these factors, only Hounsfield unit (HU) measurements obtained from computed tomography (CT) provide an indication of stone hardness. Consequently, HU is widely used to predict ESWL success, especially in adult patients [5]. However, patients with a history of kidney stones often undergo multiple CT examinations, resulting in a high cumulative radiation dose [6]. Given the longer life expectancy and increased radiation sensitivity in pediatric patients, the use of CT is limited [7]. Therefore, development of alternative, radiation-free methods to assess stone hardness without relying on CT is necessary.

Shear wave elastography (SWE) is a radiologic technique that utilizes ultrasonography (US) to visualize and quantify tissue stiffness [8]. In a previous pilot study on adults, we demonstrated that SWE could be used to predict the success of ESWL and serve as an alternative to HU [9]. In the present study, we aimed to evaluate the applicability of SWE in predicting ESWL outcomes in a pediatric patient population, for whom radiation exposure is a greater concern. To our knowledge, this is the first study to assess the use of SWE for predicting ESWL success in children.

## Materials and methods

**Fig. 1:**
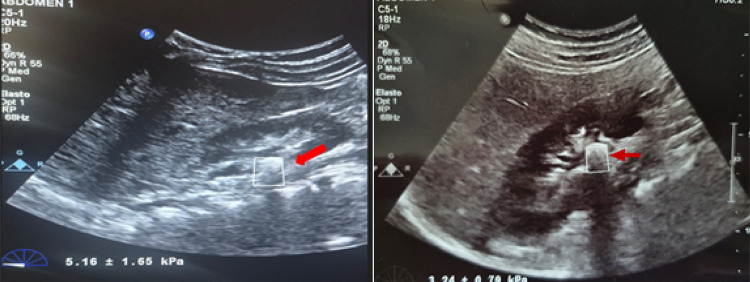
SWE measurement of renal stone

This prospective study was initiated after obtaining approval from the Committee of our University Faculty of Medicine (HRU/21.08.03). Pediatric patients under the age of 18 years who presented to our outpatient clinic between May 2021 and December 2023 with a diagnosis of kidney stones and were followed up for at least 3 months were included in this study. ESWL was planned for patients with stone sizes in the range of 6–20 mm who consented to treatment. For patients who consented to participate in the study, stone elastography values were measured by a single radiologist using grayscale renal US and SWE. Written informed approval was obtained from the legal representatives of all patients before the procedure. SWE measurements were performed once prior to ESWL. A Philips EPİQ7 system (Philips Medical Systems, Bothell, WA) with a 1–5-MHz abdominal probe was used for the procedure. Stone localization was determined using US examination while the patients were in supine and lateral decubitus positions. Subsequently, SWE measurements of the stone was obtained in kilopascals (kPa) (Fig. 1).

Patients with internal stent, ureteral stones, or abnormal renal anatomies (pelvic kidney, horseshoe kidney, and rotational anomaly) were excluded from the study. Age, sex, stone location, stone size (maximum longitudinal and transverse dimensions), BMI, and stone elastography values were recorded for all included patients. HU values were documented for 34 patients who had undergone CT during the diagnostic phase. ESWL was performed under sedation and analgesia (midazolam: 0.05–0.1 mg/kg, ketamine: 1 mg/kg). ESWL was performed at 14–21 kilovolts (kV), at a rate of 80 shocks per minute, with 1800 shocks per session. In all cases, the power was gradually increased, starting from lower values. All patients were monitored for vital signs and potential cardiac arrhythmias, and appropriate fluid replacement was administered. The participants were kept under observation for 2 h after the procedure. ESWL was performed using an Elmed device (Elmed Medical Systems Multimed Classic, Turkey). Following each session, stone fragmentation was evaluated during the first week using X-ray and US. In cases with fragments measuring > 5 mm, ESWL was repeated at intervals of at least 15 days, with a maximum of three sessions. All patients were evaluated using X-ray and US 3 months after the final ESWL session. The absence of stone fragments on imaging as well as the presence of clinically insignificant fragments < 4 mm that did not cause urinary infection or obstruction were considered indicative of a positive response to ESWL. The patients were subsequently categorized into two groups: responders and nonresponders, and stone elastography values along with other parameters were compared between the groups. The correlation between HU and SWE was examined in 34 patients who had undergone CT at the diagnosis stage. Sample size calculation was performed using PASS 11 software, with a power of 99% and a significance level (alpha) of 0.05. Based on the findings of our previous study [9], a minimum of 50 patients with kidney stones was determined to be necessary to meet the study objectives.

## Results

Of the 102 patients included in this study, 45 were female and 57 were male. The mean age of the patients was 6.5 (7 ± 4.1) years. The mean stone size was 12 (12.5 ± 3.5) mm, and the mean stone SWE value was 11.9 (12.8 ± 4.1) kPa (Table 1).

**Table 1:** Patients’ characteristics

	Min–Max	Median	Mean ± sd/*n*-%	
Age	1.0– 17.0	6.5	7.0 ± 4.1	
*Gender*				
Male			57	55.9%
Female			45	44.1%
BM-İndex (kg/m^2^)	17.3– 37.9	27.4	27.1 ± 3.8	
*Side*				
Right			51	50.0%
Left			51	50.0%
*Localisation*				
Lower Pole			13	12.7%
Middle Pole			20	19.6%
Pelvis			55	53.9%
Upper Pole			14	13.7%
Stone Size (mm)	7.0– 20.0	12.0	12.5 ± 3.5	
SWE (kPa)	6.8– 26.2	11.9	12.8 ± 4.1	
*Number of ESWL Sessions*				
I			30	29.4%
II			47	46.1%
III			25	24.5%
BT Hounsfield	402.0– 1408.0	976.5	969.8 ± 234.3	
*Responders*				
(–)			24	23.5%
(+)			78	76.5%

BMI; body mass index. ESWL; extracorporeal shock-wave lithotripsy. SWE; share wave elastography

Among the 102 patients included in this study, 78 exhibited a positive response to ESWL. No significant differences in age, sex distribution, and laterality were observed between the responder and nonresponder groups (*p* > 0.05). However, the responder group had a significantly higher BMI than the nonresponder group (*p* < 0.05) (Table 2). Furthermore, in the responder group, the SWE values, HU, stone sizes, and lower pole localization were significantly lower than those observed in the nonresponder group (*p* < 0.05) (Table 2).

**Table 2:** Comparison of the patient groups

	Non-Responders (*n*:24)			Responders (*n*:78)			*p*
	Mean ± sd/*n*-%		Median	Mean ± sd/*n*-%		Median	
Age	7.0 ± 4.0		6.0	7.0 ± 4.2		7.0	0.959^m^
*Gender*							
Male	13	54.2%		44	56.4%		0.847^X²^
Female	11	45.8%		34	43.6%		
BM-İndex (kg/m^2^)	25.5 ± 3.3		25.3	27.7 ± 3.8		27.9	***0.015*** ^m^
*Side*							
Right	12	50.0%		39	50.0%		1.000^X²^
Left	12	50.0%		39	50.0%		
*Localisation*							
Lower Pole	6	25.0%		7	9.0%		***0.040*** ^X²^
Middle Pole	2	8.3%		18	23.1%		***0.037*** ^X²^
Pelvis	11	45.8%		44	56.4%		0.363^X²^
Upper Pole	5	20.8%		9	11.5%		0.247^X²^
Stone Size (mm)	13.8 ± 3.3		14.0	12.1 ± 3.5		12.0	***0.032*** ^m^
SWE (kPa)	18.6 ± 3.4		18.6	11.0 ± 2.2		10.7	***0.000*** ^t^
*Number of ESWL Sessions*							
I	7	29.2%		23	29.5%		0.473^X²^
II	9	37.5%		38	48.7%		
III	8	33.3%		17	21.8%		
BT Hounsfield	1205.5 ± 143.3		1208.5	871.6 ± 191.1		887.0	***0.000*** ^t^

Statistically signifcant results are in bold italics (*p* < 0.05)

^m^Mann-whitney u test / ^X²^Chi-square test (Fischer test) / ^t^Independent Samples t test

**Fig. 2:**
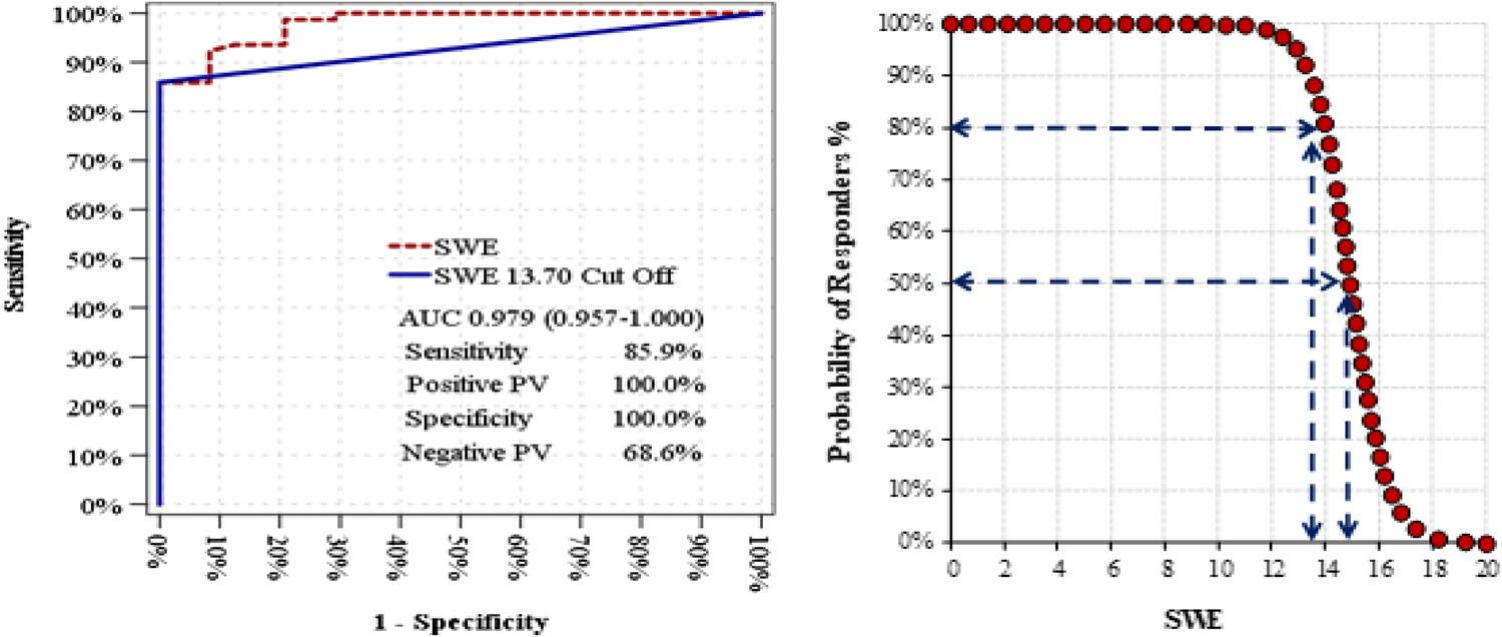
ROC Curve

The stone SWE value was 10.7 kPa (11 ± 2.2) in the responder group and 18.6 kPa (18.6 ± 3.4) in the nonresponder group. SWE exhibited significant efficacy in distinguishing between ESWL responders and nonresponders, with an area under the curve (AUC) of 0.979 (0.957–1.000). An optimal SWE cutoff value of 13.70 kPa was identified for patient differentiation, yielding an AUC of 0.929 (0.881–0.978). At the SWE cutoff value of 13.7 kPa, the sensitivity, positive predictive value, specificity, and negative predictive value for distinguishing ESWL responders from nonresponders were 85.9%, 100.0%, 100.0%, and 68.6%, respectively (Fig 2).

**Fig. 3:**
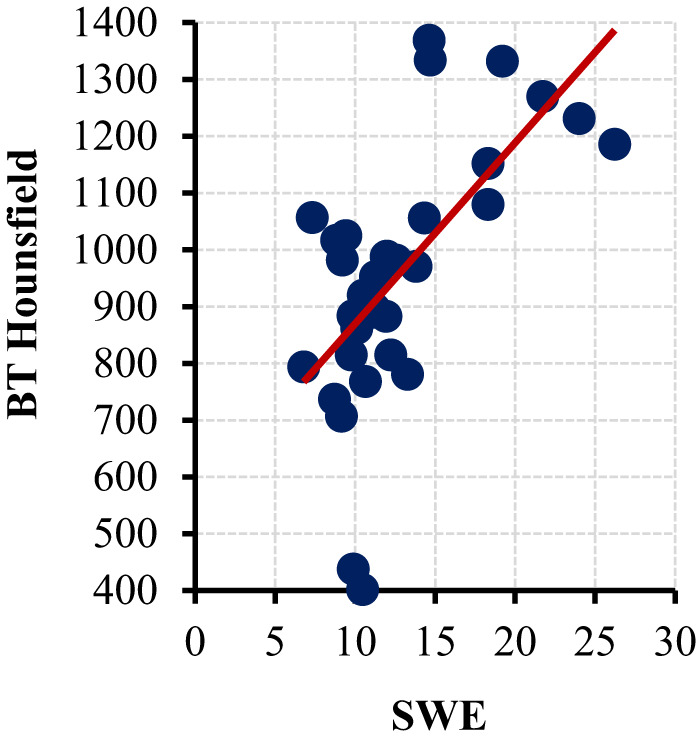
SWE and HU Pearson Correlation

A statistically significant positive correlation was observed between the SWE values and CT HU values (*p* < 0.05) (Fig 3).

## Discussion

In this study, we evaluated the efficacy of using SWE to predict the success of ESWL in pediatric patients. The findings indicate that SWE may serve as a robust predictor of ESWL responses in children. ESWL was introduced for kidney stone treatment in 1980 and subsequently used in pediatric patients by Newman et al. in 1986 [10]. Shock wave energy is transmitted more effectively in children due to the shorter skin-to-stone distance, thereby enhancing treatment efficacy [11]. Over the years, the high success rates and low complication rates reported in pediatric cases have established ESWL as one of the preferred treatment methods [12]. Nevertheless, the necessity for repeated sessions can impose additional stress on both the child and family as well as increase the risk of anesthesia-related complications, which are among the major disadvantages of the procedure [13]. Consequently, the ability to predict ESWL success preoperatively is crucial.

The literature indicates that the stone-free rates after a single ESWL session in children range from 43.8 to 82.4% (4, 5), whereas repeated sessions can increase the success rates between 70% and 90% [5, 14]. Numerous factors have been identified that affect ESWL success [4, 5]. Stone composition and HU measurement using CT provide an estimate of stone hardness. In routine clinical practice, HU is commonly used due to the lack of pre-ESWL stone analysis. Several studies have demonstrated that HU is an independent predictor of ESWL success in adults [15, 16]. Ouzaid et al. reported stone-free rates of 38% in patients with HU > 970 and 96% in those with HU < 970 [17], whereas Hameed et al. demonstrated that stone fragmentation decreased in patients with HU > 1350 [18]. In a study by Massoud et al., patients were categorized into three groups based on HU values (> 1000, 500–1000, and < 500) and their ESWL success rates were 44.6%, 95.7%, and 100% respectively [19].

CT imaging in pediatric patients exposes them to a considerable amount of radiation. Even with low-dose CT protocols, pediatric patients may be exposed to up to 0.5 mSv of radiation [20, 21]. Given the recurrent nature of urolithiasis, multiple CT examinations result in an increased cumulative radiation dose [6]. Due to these concerns, there are only a limited number of studies examining the predictive value of HU for ESWL success in children. Mc Adams et al. divided pediatric patients into two groups based on HU (< 1000 and > 1000) and reported ESWL success rates of 77% and 33%, respectively [22]. In a study by El-Assmy et al., patients were divided into two groups based on HU (≤ 600 and > 600), and ESWL success rates were reported as 82.1% and 20%, respectively [23]. Akıncı et al. compared pediatric patients who had undergone ESWL with and without pretreatment CT and found comparable stone-free rates in both groups. The authors advocated for a reduction in CT usage to minimize radiation exposure in children [20]. In our clinic, we do not routinely perform CT scans in pediatric patients to predict ESWL success due to radiation exposure. However, CT scans are performed on some patients in the emergency room or in our clinic to exclude other pathologies during the diagnosis phase. In the present study, only 34 of the 102 patients had undergone CT during the diagnostic phase. The mean HU value was 887 in the ESWL responder group and 1208.5 in the nonresponder group.

SWE measures tissue stiffness by transmitting short-duration, high-frequency sound waves to the tissue using ultrasound probes and calculating the resulting shear wave speed. Because this measurement technique is not operator-dependent, the results are considered to be objective [8]. In a study by Kraev I et al., SWE was shown to be useful for quantifying the hardness of kidney stones for the first time [24]. In a previous pilot study on adult patients, we were the first to report that SWE can be used to predict the success of ESWL and that SWE and HU are correlated. In that study on 52 adult patients, the average stone SWE value was 7.3 kPa in ESWL responders and 14.6 kPa in nonresponders [9]. Subsequently, Samir et al. reported an SWE value of 11.74 ± 3.86 kPa in the ESWL responder group and 17.51 ± 3.07 kPa in the nonresponder group. They identified a cutoff value of 15.5 kPa for predicting ESWL success and concluded that SWE could serve as an alternative to HU [25]. In the present study conducted on pediatric patients, the stone mean SWE value was 10.7 (11.0 ± 2.2) kPa in responders and 18.6 (18.6 ± 3.4) kPa in nonresponders. An SWE cutoff value of 13.70 kPa was calculated for predicting ESWL success. This difference was highly statistically significant (*p* < 0.05). When the 34 patients who had undergone CT were evaluated separately, a correlation was observed between SWE and HU, consistent with previous studies [9, 25] (Fig. 3). These findings indicate that SWE is a reliable method for assessing stone hardness and possesses a high predictive value for forecasting ESWL success.

When additional factors affecting ESWL outcomes were examined, BMI emerged as an important parameter. Studies conducted in adults have reported that in obese patients, the energy transmitted to the stone is reduced, resulting in lower stone-free rates [26]. Moreover, skin-to-stone distance has been identified as a critical factor in predicting ESWL success [27, 28]. However, studies in pediatric populations have not consistently shown a significant effect of obesity on ESWL outcomes [22, 29]. In contrast, Kızılay et al. reported lower ESWL success rates in obese children. The authors attributed this discrepancy to the broad age distribution of their patient cohort [30]. In the present study, contrary to previous findings, higher BMI was associated with increased ESWL success. We hypothesize that this may be due to reduced kidney mobility in overweight children as a result of increased perirenal adipose tissue, which could result in a favorable response to ESWL.

This study had several limitations. First, it had a relatively small sample size, which may limit the generalizability of the results. Second, SWE measurements were performed at a single center by one operator, which may have reduced interobserver variability. Multicenter studies are necessary to confirm the validity of these results in a larger patient population. Another limitation is the lack of stone composition analysis in the patients. Studies comparing SWE values according to stone types are needed.

In conclusion, the present study demonstrates that SWE is an effective method for predicting ESWL success in pediatric patients. As shown in both our previous studies and the present study, SWE and HU are significantly correlated. Compared with CT, SWE offers a cost-effective, radiation-free, and easily applicable alternative for assessing stone hardness, contributing significantly to clinical practice. Future large-scale, prospective studies are warranted to further elucidate the clinical utility of SWE in predicting ESWL outcomes.

## Data Availability

No datasets were generated or analysed during the current study.
